# Determining crossover count and position in two pig lines with different selection histories

**DOI:** 10.1186/s12711-026-01060-x

**Published:** 2026-06-20

**Authors:** Olivia Katz, Anne C.M. Jansen, Tom Druet, Anne Boshove, Mario P.L. Calus, Yvonne C.J. Wientjes

**Affiliations:** 1 Animal Breeding and Genomics, Wageningen University and Research, Wageningen, 6700 AH The Netherlands; 2 Unit of Animal Genomics, Faculty of Veterinary Medicine, GIGA-R &, University of Liège, Avenue de l’Hôpital, 1, Liège, 4000 Belgium; 3 Topigs Norsvin Research Center B.V, 5216 TZ ’s-Hertogenbosch, the Netherlands

## Abstract

**Background:**

Crossovers are crucial in meiosis and can break up the link between neighboring loci, creating novel allele combinations and genetic variation. However, their potential use in livestock breeding programs remains unexplored. Here, we studied the patterns of crossover count in two pig lines with different selection histories. We analyzed genomic data from over 47,000 individuals in a boar line and 78,000 individuals in a sow line, using over 20,000 autosomal single nucleotide polymorphisms (SNPs). We estimated and compared autosomal crossover counts (ACC), intrachromosomal genetic shuffling, and inter-crossover distances when two crossovers are transmitted on a chromosome. Finally, we compared the recombination landscapes of individuals transmitting a high versus a low number of crossovers.

**Results:**

The mean ACC was 19.9 (SD: 4.7) for the boar line and 20.9 (SD: 5.0) for the sow line. On all chromosomes and in both lines, 0, 1 or 2 crossovers were transmitted in > 93% of the cases. In the sow line, parents transmitted nearly one additional crossover and exhibited slightly higher overall intrachromosomal genetic shuffling than in the boar line in both sexes. Dams generally transmitted more crossovers than sires in both lines. Particularly when transmitting two crossovers, intrachromosomal shuffling was higher in sires than in dams, except for the five shortest chromosomes, suggesting that sires space crossovers more evenly across chromosomes. Sires also showed greater mean inter-crossover distances on all chromosomes, except for *Sus scrofa* chromosomes 1, 13, and 18, which may reflect less potential to release genetic variance in the shortest chromosomes. Lastly, individuals with the highest crossover count more often localize crossovers near chromosome centers than those with the lowest counts, which might generate more genetic variance towards center regions.

**Conclusions:**

Our results confirm that dams transmitted more crossovers than sires in both lines. Additionally, crossover counts were higher in the sow line, possibly due to different selection histories. Individuals with the most transmitted crossovers localized more often crossovers towards chromosome centers. The different patterns of crossover counts can generate different levels of genetic variance, which might be important to consider before applying selection on crossover count in commercial breeding programs.

**Supplementary Information:**

The online version contains supplementary material available at 10.1186/s12711-026-01060-x.

## Background

During meiosis, homologous chromosomes can undergo a process known as recombination. A recombination can have two possible outcomes: a crossing-over or a non-crossing over. While non-crossing overs refer to unilateral transfer of DNA from one homologous chromosome to the other, crossover refers to the reciprocal exchange of DNA between homologous chromosomes [1]. By exchanging DNA, crossovers can create novel combinations of alleles in populations by breaking linked loci, which can result in more genetic variation [2]. This creates advantages for adaptation of species to changing environments [3], the maintenance of genetic diversity [4, 5] and potentially also for animal breeding purposes [2, 4].

The crossover events that take place during the formation of gametes are independent between different chromosomes. However, crossover events don’t occur randomly across the genome. The number and frequency of crossovers can be influenced by multiple factors, such as the sex, breed and species of the individual [6, 7], the genomic region, and processes like crossing-over interference, a phenomenon where the occurrence of a crossover at one position decreases the likelihood of additional crossover happening nearby on the same chromosome [5]. Across species, males and females often differ in their recombination rate, a phenomenon known as heterochiasmy [1, 8]. For example, in species like cattle and sheep [1, 9], males tend to recombine more than females. However, in species like the mouse, the Atlantic salmon and the human, females have a higher overall recombination rate, except for the telomere region [10–12], where recombination rate is higher in males [13, 14]. In pigs, females also have a higher recombination rate [6, 7, 15], but this is even the case in the telomere region [7, 14]. Moreover, studies have reported that in pigs, both sexes show a recombination landscape marked by higher rates near the telomeric ends of chromosomes and lower rates in the centromeric regions [7, 15]. In addition, another factor associated with recombination rate is the content of GC nucleotides, which was found to have a stronger correlation with recombination rate in females than in males [6]. Furthermore, age affects recombination rate, and dams with a higher parity number show higher recombination rates towards their offspring [16].

Although sex differences in the pig recombination landscape have been reported [7, 14], to our knowledge no study in any species—including pigs—has examined potential differences in recombination landscape between individuals transmitting high and low crossover numbers. Since breeding goal traits are quantitative and controlled by many unknown polygenes, crossovers near the regions containing the genes may generate new additive genetic variance. Thus, for animal breeding, differences in the recombination landscape might be important, as crossover positions may affect the release of additive genetic variance in breeding goal traits.

The lack or absence of crossover events can result in problems during meiotic segregation of homologous chromosomes [17]. This can lead to the production of non-disjunction events and aneuploid gametes [18, 19], which can reduce fertility [20–22]. Beside the direct impact of recombination rate on aneuploidy and fertility, a lower recombination rate can also indirectly impact fertility due to the increased expression of deleterious alleles [23]. Thus, it is expected that a certain level of recombination rate is required to maintain a fertile population. However, a very high recombination rate could increase the chances for detrimental mutations and also cause recombination load, a mechanism that breaks up favorable combinations of alleles [5]. These observations suggest that there may be an optimal recombination rate for fertility performance. The impact of recombination rate on fertility might suggest that artificial selection on fertility could change the recombination rate to reach the optimal recombination rate for fertility performance. We hypothesize that this difference in recombination rate can be studied by comparing two pig lines with different selection histories: a sow line in which selection is mainly aimed at improving fertility performance, and a boar line in which selection is aimed at productivity traits. Under this hypothesis, differences in recombination rate between the two lines are expected, reflecting their distinct selective histories.

In addition, there is individual variation in the number of crossovers that parents transmit to their offspring [15]. Heritability estimates of recombination rates in pigs range between 0.07 and 0.11 in females and between 0.04 and 0.05 in males [7, 15]. A metric that describes the impact of the location of crossovers is intrachromosomal genetic shuffling, which reflects the likelihood that the alleles at two randomly selected loci shuffle during meiosis as a consequence of crossovers [24]. It is dependent on the recombination rate, the position of the crossover events, and is maximized when crossovers events are evenly distributed across a chromosome [24], which implies that each chromosome contains 50% of each paternal (or maternal) grandparent’s information. A higher degree of intrachromosomal genetic shuffling during meiosis leads to increased genetic diversity in the creation of gametes. Intrachromosomal genetic shuffling was also found to be heritable in pigs, with a heritability of 0.04 in males and 0.09 in females [25]. These results imply that it should be possible to either influence the recombination rate, as well as the level of genetic shuffling by means of selection.

This study aimed to: (1) estimate and compare autosomal recombination rates and ACC between two commercial pig lines with different selection histories; (2) analyze and compare the recombination landscape between individuals with a high and low number of transmitted crossovers; (3) estimate and compare the individual genetic shuffling across all autosomes; and (4) estimate the distances between consecutive autosomal crossover events in both pig lines. In this study, we used genomic data from over 20,000 autosomal SNPs of 91,371 parent-offspring pairs of a boar line and 150,228 parent-offspring pairs of a sow line. These parent-offspring pairs were extracted from two initial datasets containing over 120,000 individuals of the boar line and over 240,000 individuals of the sow line.

## Methods

### Selection of target individuals in the datasets

Data was provided by the pig breeding company Topigs Norsvin and included two commercial pig lines: a synthetic boar line comprising 128,667 individuals, and a Large White sow line comprising 246,630 individuals. For all animals, 23k Single Nucleotide Polymorphisms (SNPs) genotype data from an Illumina Geneseek custom 25 K SNP chip was available. SNP positions were mapped to the Sscrofa11.1 reference genome assembly. All SNPs from the sex chromosome were excluded from the analysis.

As a first step, some quality control (QC) filters were applied to individuals and SNPs in the initial dataset. In this step, individuals with more than 1% missing genotypes and SNPs with more than 1% missing genotypes were excluded. Subsequently, we retained only families for which at least five full sib (FS) offspring, both parents, and all four grandparents were genotyped. This filter was applied to ensure comparable information across families and to accurately infer the parental haplotype origin.

After filtering, the final datasets consisted of 5,642 families in the boar line with 47,549 unique individuals and 8,265 families in the sow line with 78,446 unique individuals. All individuals were genotyped for 20,713 SNPs. An overview of the workflow leading to the final datasets is presented in Additional file 1 Fig. S1. Crossover events were inferred from parent–offspring pairs, such that each parent–offspring pair corresponded to one observation of crossovers in the transmitting parent. Details about the pedigrees, families and final datasets are presented in Table 1.

### Identifying crossovers

Using the genomic data, we tracked chromosome segments across generations to identify autosomal crossovers in three steps (Additional file 1 Fig. S2). The first step involved phasing the genotypes and detecting the parental origins of the haplotypes. The phasing was done with the program LINKPHASE3 [26] (downloaded from https://github.com/tdruet/LINKPHASE3) using 1 iteration, which required positions for all SNPs in centimorgan (cM). For this, we used a physical map based on base pair positions, and we translated those positions to cM using an initial value of 1 cM/Mb to start the iterative algorithm [26]. This initial value is only used to start the algorithm, which then estimates the genetic map based on the pedigree and genotype information.

Then, conflictive parent-offspring pairs with more than 50 opposing homozygotes or Mendelian errors were removed. For all 96 cases in the boar line and 334 cases in the sow line, the respective parent or parents were set to missing in the pedigree. As a result, there were 49 (boar line) and 94 (sow line) unique sires and 55 (boar line) and 120 (sow line) unique dams of individuals with relationship removed from the pedigree. Also, SNPs with > 5 Mendelian errors were excluded, resulting in the exclusion of 169 SNPs in the boar line and 290 SNPs in the sow line, leaving 20,544 SNPs in the boar line and 20,423 SNPs in the sow line.

As a second step, LINKPHASE3 [26] was run with 1 iteration to verify that each parent-offspring pair had under 50 Mendelian errors. Then, we analyzed possible map errors (i.e. incorrect SNP positions) through LINKPHASE3’s output file Map Confidence Score (MCS). The MCS of each SNP indicates the confidence that the SNP position is correct on the linkage map [26]. For the boar line, evidence for map errors occurred on SSC (SSC) 3 (two SNPs) and 5 (one SNP). In the sow line, map errors were found on SSC 1 (one SNP), 3 (three SNPs), 5 (one SNP, the same SNP with evidence for map error in the boar line), 7 (one SNP), and 14 (two SNPs). Therefore, we decided to manually exclude those SNPs from those chromosomes in the line in which the map error occurred. However, for the SNP with a low MCS on SSC 5, removing the SNP was not enough to remove the mapping error and a more detailed analysis was needed (as presented in the Results section). Specifically, for SSC 5, there was a peak in recombination rate between the two SNPs positioned at 11.8556 Megabase (Mb) and 12.1131 Mb in both lines. This could suggest a flipped chromosome region, where the region until the SNP located at 11.8556 Mb was reversed compared to the reference map. To investigate whether this was the case, we re-run LINKPHASE3 [26] with the first region flipped, which resulted in even higher recombination rates. This indicates that the original orientation was correct. As a result, in total 20,541 SNPs remained in the boar line, and 20,415 SNPs in the sow line.

As a third step, LINKPHASE3 [26] was rerun using the cleaned pedigree and SNP dataset to estimate the number of crossovers by chromosome for parent-offspring pairs of both pig lines. This run included the estimation of the sex specific genetic maps (with the number of iterations set to 50). After haplotype phasing, LINKPHASE3 infers crossovers by analyzing inheritance patterns between parent-offspring pairs. The number of crossovers by chromosome per parent-offspring pair is extracted directly from the output and used to estimate the ACC. The ACC was defined as the number of crossover events across all autosomes that a parent transmitted to an offspring. Thus, each offspring received two parental ACC estimates, one from its sire and one from its dam. The total number of parent-offspring pairs with ACC estimates were 91,371 for the boar line and 150,228 for the sow line and the total number of identified crossovers per sex and line are in Table 1.

**Table 1:** Description of the two datasets after quality control

Line	Boar line	Sow line
Number of families	5,642	8,265
Number of dam-offspring pairs after quality control	45,182	74,223
Number of sire-offspring pairs after quality control	46,189	76,005
Number of total parent-offspring pairs after quality control	91,371	150,228
Number of identified crossovers in dam-offspring pairs	1,012,523	1,771,355
Number of identified crossovers in sire-offspring pairs	803,691	1,374,729
Number of male offspring	25,733	33,838
Number of female offspring	19,979	42,832
Number of offspring with unknown sex	1,837	1,776
Number of unique sires	588	840
Number of unique dams	4,891	7,776

We assumed that crossovers on different chromosomes were independent from each other. To detect possible outliers for crossover count on each particular chromosome, we compared the distribution of observed crossover count reported by LINKPHASE3 with the expected crossover count values assuming a Poisson distribution with λ set to the observed crossover count mean [27] of each chromosome (Additional file 1 Fig S3). No outliers were excluded because the observed frequencies of high crossover count were consistent with the expected probabilities, showing no significant deviations. All the statistical analysis were performed using R statistical software (v4.4.1; R Core Team 2024) [28].

### Estimation of sex-specific recombination rate

To estimate the overall recombination rate per Mb in both lines, we first estimated the genetic length of each autosome per sex as the sum of all genetic distances between consecutive SNPs in the sex specific linkage maps provided by LINKPHASE3. Then, the recombination rate (cM/Mb) was calculated as the ratio of genetic length over the physical length for each autosome, per sex and line.

### Analysis of recombination landscape between individuals transmitting high and low ACC

To compare the recombination landscapes of parents with a high and low crossover transmission, we analyzed the crossover locations of the individuals with the highest (top 10%) or lowest (bottom 10%) ACC per sex. To determine the location of a crossover, we assumed it occurred exactly in the middle of the two SNPs reported by LINKPHASE3 to flank the crossover. These midpoint positions were then used to construct recombination landscapes by calculating the relative frequency of crossovers along each autosome for males and females. Frequencies were compared between the top and bottom 10% groups to identify potential differences in recombination patterns.

### Estimation of genetic shuffling

Intrachromosomal genetic shuffling ($\:\stackrel{-}{r}$) represents the estimated probability that the alleles of a randomly chosen pair of loci are shuffled during meiosis due to crossover events. It was estimated per individual as proposed by Veller et al., 2019 [24]:

1 $$\:E\left(\stackrel{-}{r}\right)=\sum\:_{k=1}^{n}2{p}_{k}\left(1-{p}_{k}\right){L}_{k}^{2}$$

where *k* refers to the autosomes 1 to 18; $\:{p}_{k}$ is the probability that two random loci on chromosome *k* come from a different parental origin and was estimated based on the origins_hmm.txt outputs of LINKPHASE3, which indicates the origins of the phased genotypes; and $\:{L}_{k}$ is the fraction of the genome’s length in Morgan associated with chromosome *k*. These estimates provided individual-level phenotypes of intrachromosomal genetic shuffling. Additionally, line and sex-specific estimates were obtained by averaging the individual estimates for intrachromosomal genetic shuffling within each group. Besides estimating the total intrachromosomal genetic shuffling, we also estimated intrachromosomal genetic shuffling for each particular chromosome, by setting *L*_*k*_ to 1.

### Estimation of inter-event distance

We also estimated per individual the physical distance in Mb between two consecutive crossovers on the same chromosome, which we refer to as the inter-event distance. For this, we again considered the position of a crossover in Mb as the midpoint between SNPs flanking a region with an observed crossover, and the inter-event distance was calculated as the difference between crossover positions on the same autosome. We included the inter-event distance in the analysis because inter-event distance is expected to be related to intrachromosomal genetic shuffling because both very short and very long inter-event distances contribute to a low amount of shuffling, while intermediate distances maximize it [24]. We restricted our analysis to the autosomes with exactly two crossovers, as for this particular case, the intrachromosomal genetic shuffling is expected to behave quadratically as a function of the inter-event distance. Thus, to maximize intrachromosomal genetic shuffling, both crossovers should be apart from each other by a distance equal to ½ chromosome length. This would be the case when $\:{p}_{k\:}=\left(1-{p}_{k}\right)$ = 0.5, in Eq. (1). Moreover, either large or short inter-event distances would be associated with lower intrachromosomal genetic shuffling values. This would be the case when$\:\:{p}_{k\:}$is higher or lower than $\:\left(1-{p}_{k}\right)$. Thus, selecting only by intrachromosomal shuffling may overlook important differences in the position of crossovers as for animal breeding purposes, short and large inter-event distances might impact the release of additive genetic variance differently, as crossovers are localized in different positions. Cases with more than two transmitted crossovers were excluded from this analysis due to their increased complexity.

## Results

### Recombination rate for both lines of pigs

The sex-specific autosomal linkage maps showed a total length of 1,879 cM (boar line) and 1,917 cM (sow line) for sires, and 2,433 cM (boar line) and 2,550 cM (sow line) for dams (Additional file 2 Table S1). This implies that the recombination rate (cM/Mb) for both sexes was slightly higher for the sow line than the boar line (sow: 1.12 cM/Mb vs. boar: 1.08 cM/Mb). In both lines, dams showed a higher recombination rate on all autosomes, except for SSC 1 and 13, which are the largest chromosomes in terms of physical length. Recombination rates also varied substantially among chromosomes, with the lowest rates observed on the largest chromosomes (SSC1 and SSC13), and the highest rates on the smallest ones (SSC12, SSC17, and SSC18).

### Autosomal crossover count

**Fig. 1:**
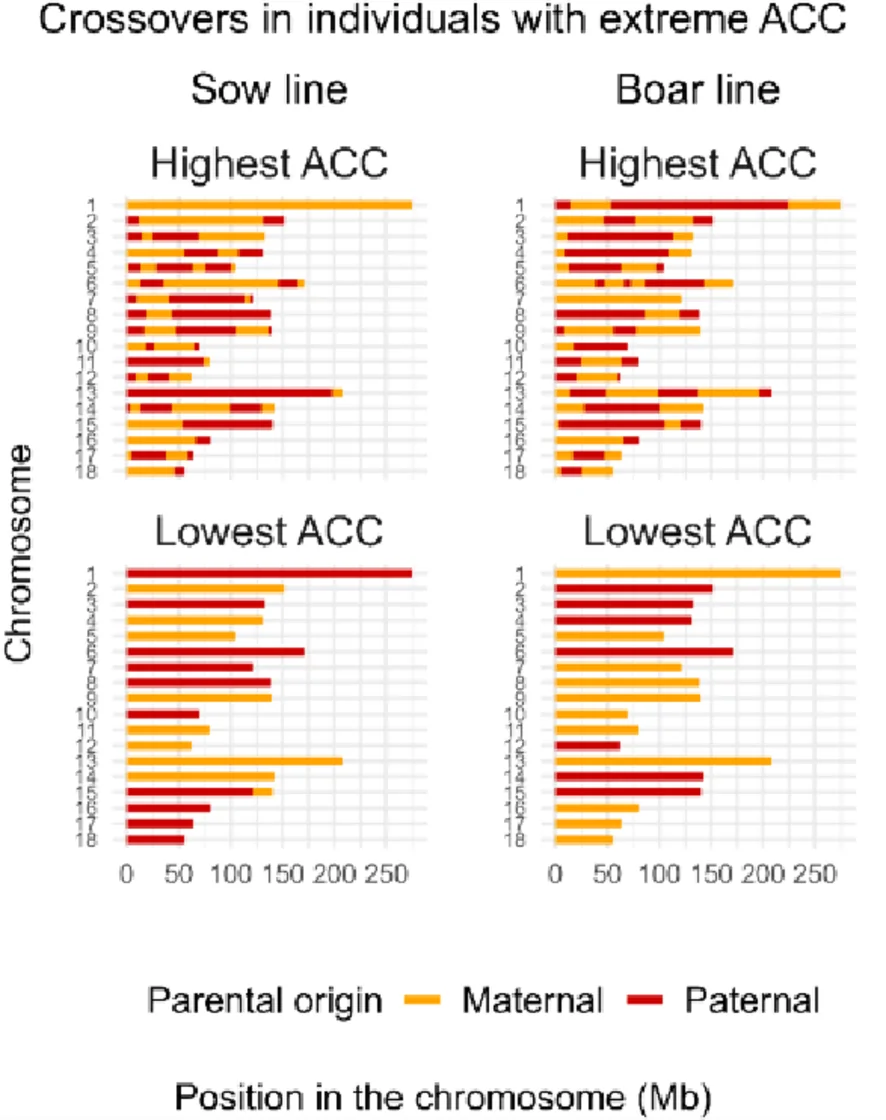
Crossovers positions for the individuals with the highest and lowest transmitted ACC in both lines

We estimated ACC values for parent-offspring pairs in the boar line and in the sow line. In both lines, dams transmitted significantly more crossovers than sires; however, the magnitude of this sex difference depended on the line, being slightly larger in the sow line. The sow line also showed significantly higher ACC than line boar in both sexes (see footnote to Table 2). On average, dams transmitted 1.29 and 1.32 times more crossovers than sires in, respectively, the boar line and the sow line (Table 2). The distributions of ACC in both lines and sexes are in Additional file 1 Fig. S4. The highest estimated ACC values were 44 and 47 for females and 33 and 34 for males, in respectively the boar and sow lines. The lowest estimated ACC values were 0 and 1 for females and 1 and 3 for males, in respectively the boar and sow lines. To illustrate these maximum and minimum ACC, Fig. 1 shows the positions of the crossovers of individuals with the highest and lowest ACC in both lines.

**Table 2:** Estimated marginal means for ACC per sex in both pig lines^a^

Line	Sex	Number of observations (number of parent-offspring pairs)	EMMs ACC (SE)	CI (95%)
Boar	Sires	46,189	17.40 (0.019)	17.36–17.44
Boar	Dams	45,182	22.41 (0.019)	22.37–22.45
Sow	Sires	76,005	18.09 (0.015)	18.06–18.12
Sow	Dams	74,223	23.87 (0.015)	23.84–23.89

^**a**^ Differences in ACC between sexes and lines were analyzed using a linear model including sex, line, and their interaction. A significant sex × line interaction was detected (*p* < 0.001).

**Fig. 2:**
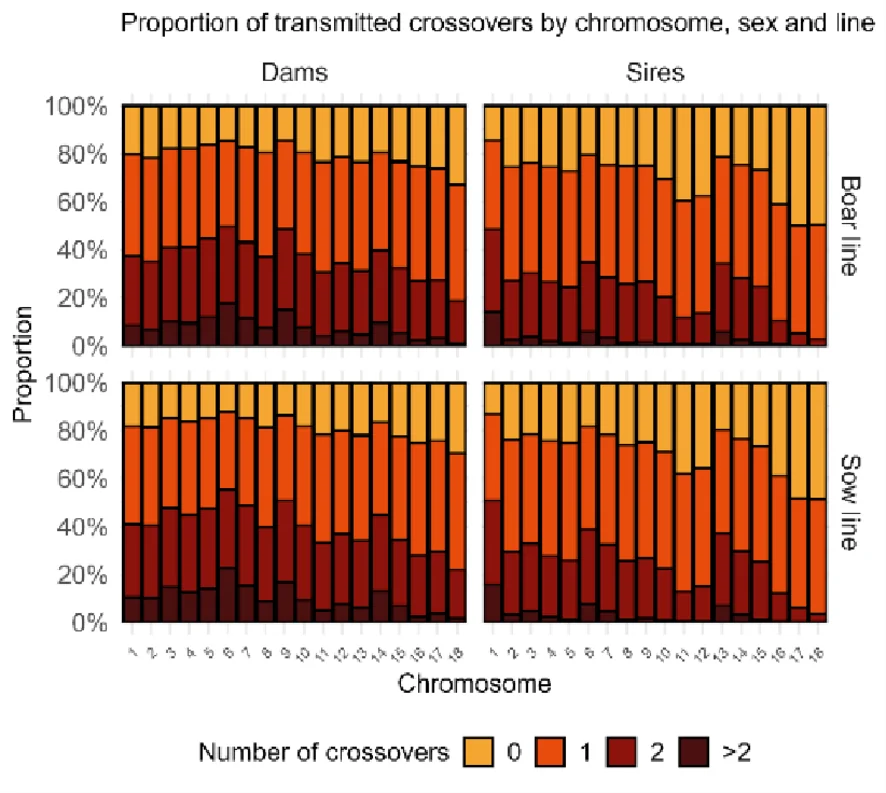
Proportion of parent-offspring pairs that transmitted 0, 1, 2 and > 2 CO per chromosome for each chromosome and by sex and line

The observed mean crossover count for each chromosome, line and sex is presented in Additional file 2 Table S2. Results indicated that larger chromosomes had a significantly higher crossover count than shorter chromosomes. Moreover, results showed that while dams generally have higher crossover count (Additional file 2 Table S2), sires tend to increase crossover count more rapidly with chromosome length, and these patterns vary subtly between lines. The proportions of parent-offspring pairs that transmitted 0, 1, 2, or more than 2 crossovers per chromosome are shown in Fig. 2 for both lines. For every chromosome, the parents most frequently transmitted 1 crossover to their offspring (44.8% boar line; 43.8% sow line). For both sexes and lines, more than 93% of the parents transmitted 0, 1, or 2 crossovers per chromosome to an offspring. Dams transmitted 3 or more crossovers more frequently than sires (on average 8.1% for dams and 1.6% for sires in boar line and 10.3% for dams and 2.1% for sires in the sow line) except for SSC 1 and 13, where the frequency was slightly higher for sires. Also, the proportion of individuals transmitting 3 or more crossovers seems to be slightly higher for the sow line than for the boar line (6.6% versus 5.2%).

### Recombination landscape for high and low recombinant individuals

To compare recombination landscapes between high and low recombinant parent-offspring pairs per line, we selected arbitrarily the top 10% and bottom 10% sires and dams in terms of ACC. This was done separately for each sex because sex-differences in the recombination landscape in pigs were previously observed [6]. In pigs, the recombination rate is not constant along the chromosomes and crossovers tend to cluster towards the chromosome ends regardless of the position of the centromere. This was observed in both sexes, with a higher effect on sows compared to boars [6, 7]. Overall, for both lines and sexes the top 10% of individuals had approximately double the crossovers than the bottom 10% of individuals (boar line top 10% sires and dams had 100 and 111% more crossovers than bottom 10%; sow line top 10% sires and dams had 96 and 104% more crossovers than bottom 10%, respectively).

**Fig. 3:**
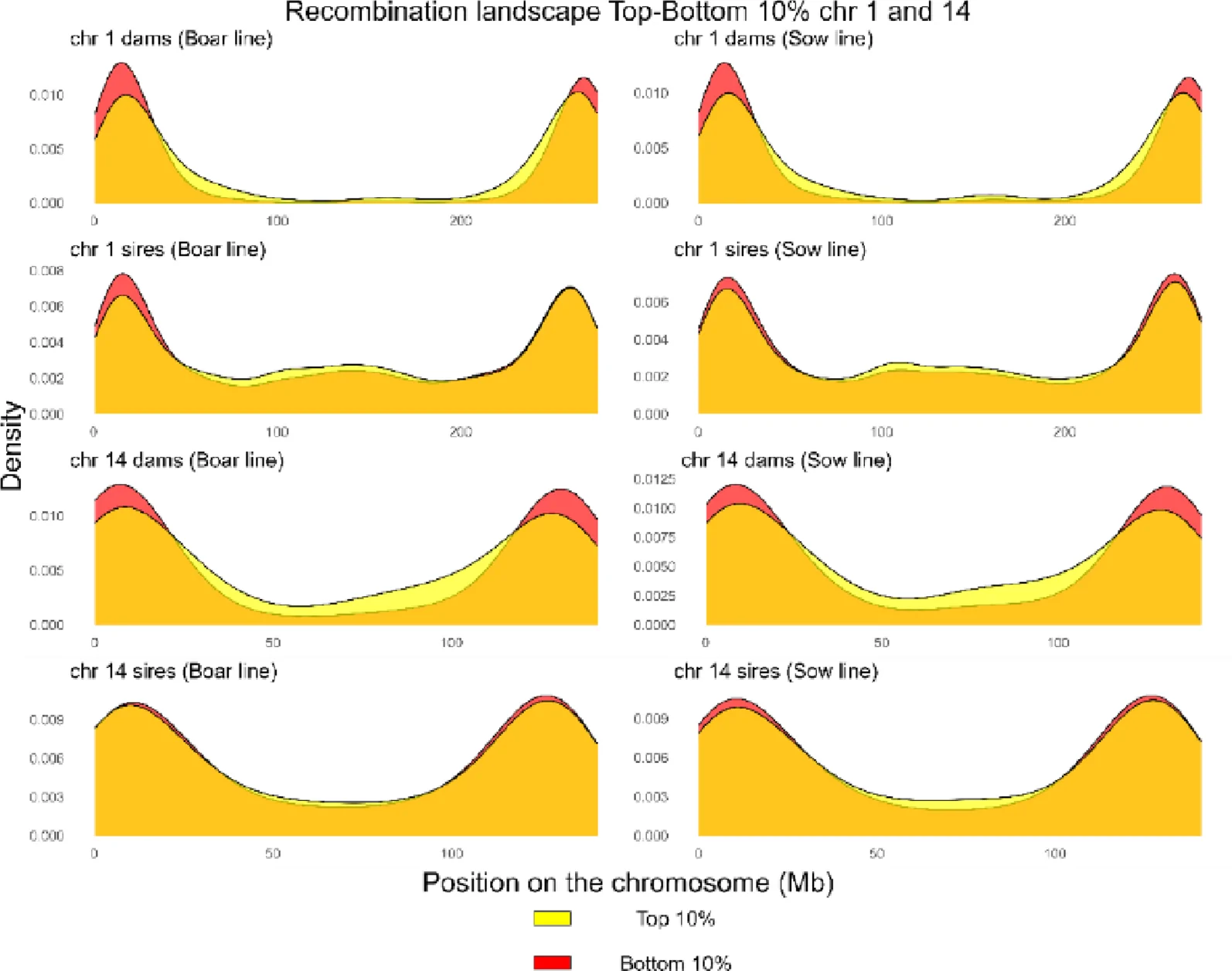
Recombination landscapes of SSC1 and 14 for top and bottom 10% of individuals with highest and lowest ACC

We compared relative frequencies in crossovers along the chromosomes between the top 10% and bottom 10% sires and dams in terms of ACC. To illustrate this, the patterns of recombination frequencies on SSC1 (metacentric chromosome [6]) and SSC14 (acrocentric chromosome [6]) are shown in Fig. 3 for both sexes and lines (all chromosomes are in Additional file 1, Fig. S5, S6, S7 and S8). The recombination landscapes were largely similar between the top and bottom 10% of individuals in terms of ACC. However, in SSC1 and SSC14, the bottom 10% showed relatively more crossovers near the telomere regions, while the top 10% showed relatively more crossovers activity toward center regions across sexes and lines. Thus, individuals with higher ACC may have slightly higher recombination rates in chromosome centers and relatively slightly lower recombination rates near the extreme chromosome regions. To formally compare the top 10% and bottom 10% of individuals based on their distribution of crossovers across the chromosomes, crossovers were classified as distal if they occurred in the first or last 25% of the chromosome and as central if they occurred in the middle 50%. The details about proportions for each group, sex and line are in Table S3 of the Additional file 2. Results indicate that the top 10% individuals not only have more crossovers per chromosome, but also that a significantly larger proportion of their crossovers occur in central regions of the chromosomes compared to bottom individuals, with the effect being slightly stronger in females.

### Intrachromosomal genetic shuffling

Individuals from the sow line exhibited 2.84% higher intrachromosomal genetic shuffling than those from the boar line (Table 3), and in both lines intrachromosomal genetic shuffling values were approximately normally distributed. Additionally, sex differences were significant in the boar line, where sires exhibited slightly higher shuffling than dams, whereas no significant sex difference was observed in the sow line.

**Table 3:** Estimated marginal means for intrachromosomal genetic shuffling in both lines and sexes^a^

Line	Sex	EMMs intrachromosomal Shuffling (SE)	CI (95%)
Boar	Sires	0.0178 (< 0.0001)	0.0177–0.0178
Boar	Dams	0.0174 (< 0.0001)	0.0174–0.0175
Sow	Sires	0.0181 (< 0.0001)	0.0180–0.0181
Sow	Dams	0.0180 (< 0.0001)	0.0180–0.0181

^a^ Differences in intrachromosomal genetic shuffling between sexes and lines were analyzed using a linear model including sex, line, and their interaction. A significant sex × line interaction was detected (*p* < 0.001).

**Fig. 4:**
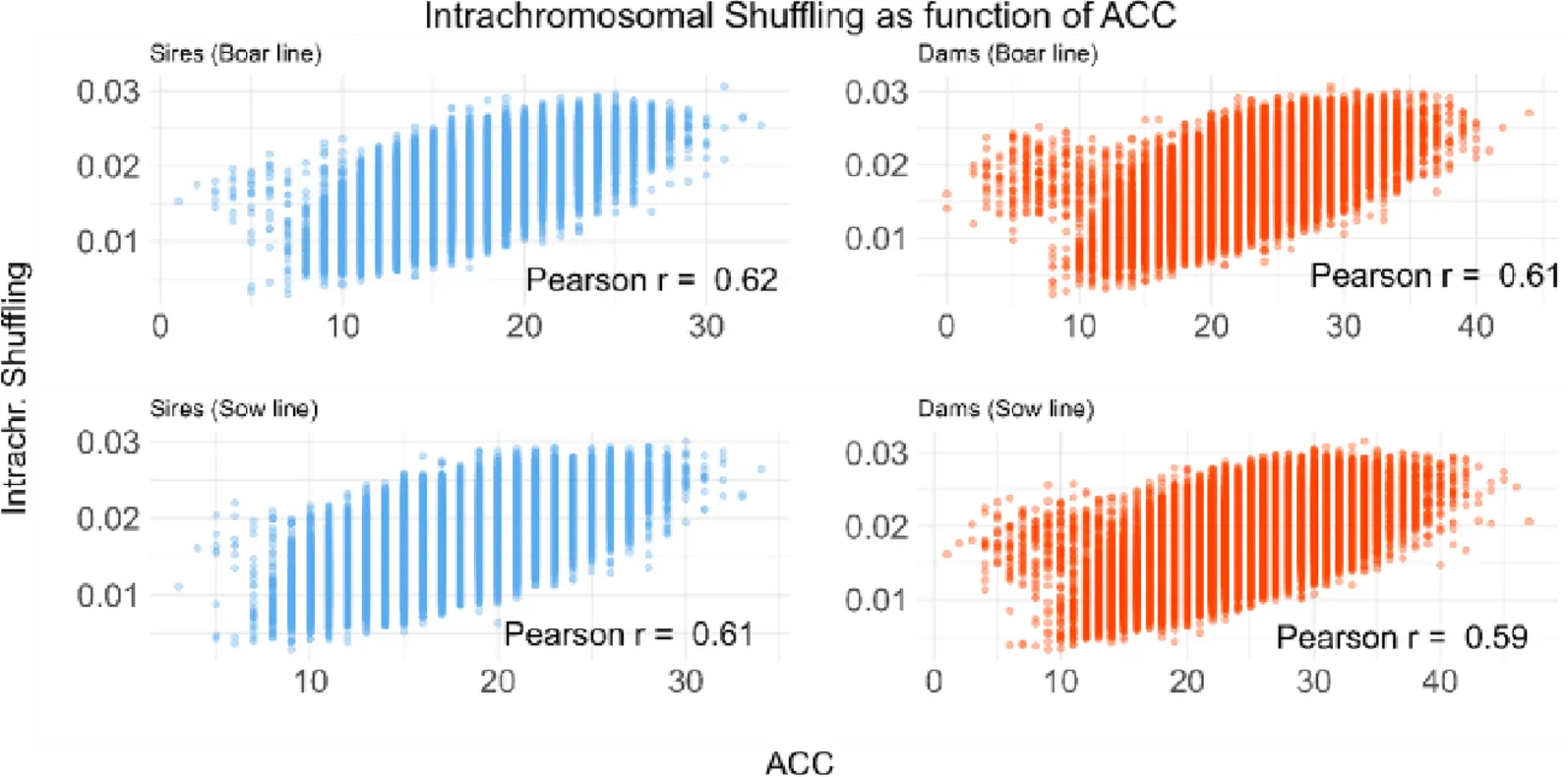
Relationship between intrachromosomal genetic shuffling and ACC in both pig lines

In both lines, Pearson correlations between intrachromosomal genetic shuffling and ACC were moderately positive (about 0.6) for both sexes (Fig. 4). Moreover, results indicate that the top 10% of dams with the highest ACC showed ~ 65% (sow line) and ~ 72% (boar line) more intrachromosomal genetic shuffling than the bottom 10%. Similarly, for sires, the top 10% showed ~ 61% (sow line) and ~ 63% (boar line) more shuffling than the bottom 10%. This indicates that, as expected, individuals with more crossovers had, on average, more intrachromosomal genetic shuffling (Additional file 2, Table S4). However, some individuals with low ACC exhibited high values of intrachromosomal shuffling (represented by the cloud of dots with ACC < 11 and intrachromosomal shuffling > 0.01 in Fig. 4). For the boar line, this group consisted of 753 parent-offspring pairs (from 495 sires and 258 dams). For the sow line, this group consisted of 664 parent-offspring pairs (from 433 sires and 231 dams). We analyzed their crossover landscape and observed a more uniform distribution of crossovers across the chromosome (Additional file 1 Fig. S9 and S10) compared to the average across all individuals. This indicates that crossovers were more evenly spaced across the chromosome, resulting in a higher intrachromosomal genetic shuffling with the same number of crossovers.

**Fig. 5:**
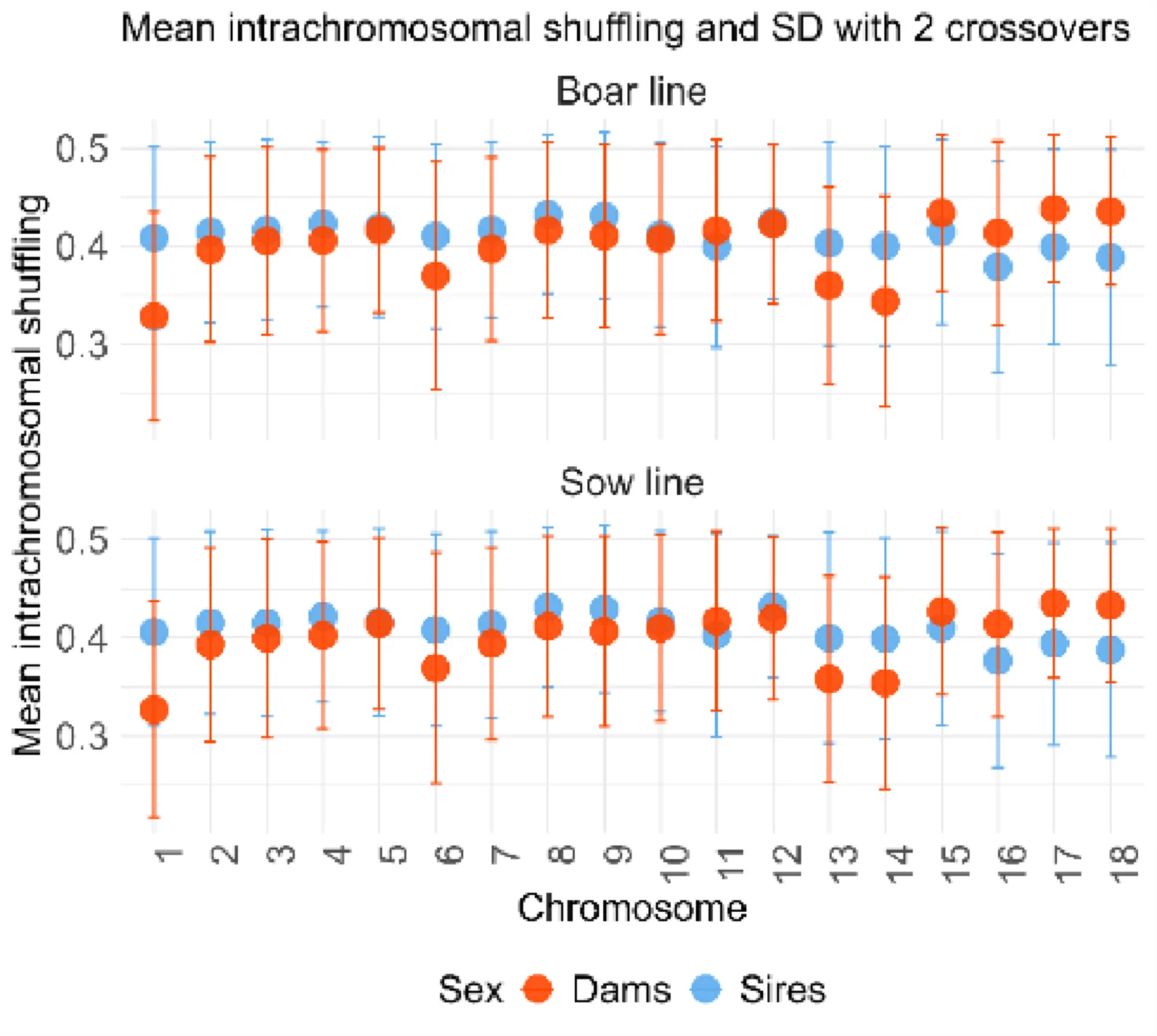
Mean intrachromosomal genetic shuffling and SD by chromosome, sex and line when the number of transmitted CO per chromosome is 2. A linear model including sex, chromosome, and their interaction was fitted to assess variation in intrachromosomal genetic shuffling, separately for each line. In both lines, the sex × chromosome interactions were highly significant (*p* < 2 × 10⁻¹⁶), indicating that sex differences in intrachromosomal genetic shuffling vary across chromosomes within each line

When focusing on the chromosomes on which two crossovers were transmitted, sires generally exhibited higher intrachromosomal genetic shuffling than dams for all chromosomes except SSC11, 15, 16, 17, and 18 (Fig. 5). These chromosomes are the shortest in terms of physical length (Mb). Therefore, significant sex differences in intrachromosomal shuffling were observed for each chromosome and number of transmitted crossovers (Fig. 5), although the observed sex-specific differences were generally small (Table 3). These results suggest that when two crossovers are transmitted on a chromosome, sires tend to localize their crossovers more evenly spaced compared to dams, except for the shortest chromosomes (Fig. 5).

### Inter-event distance

**Fig. 6:**
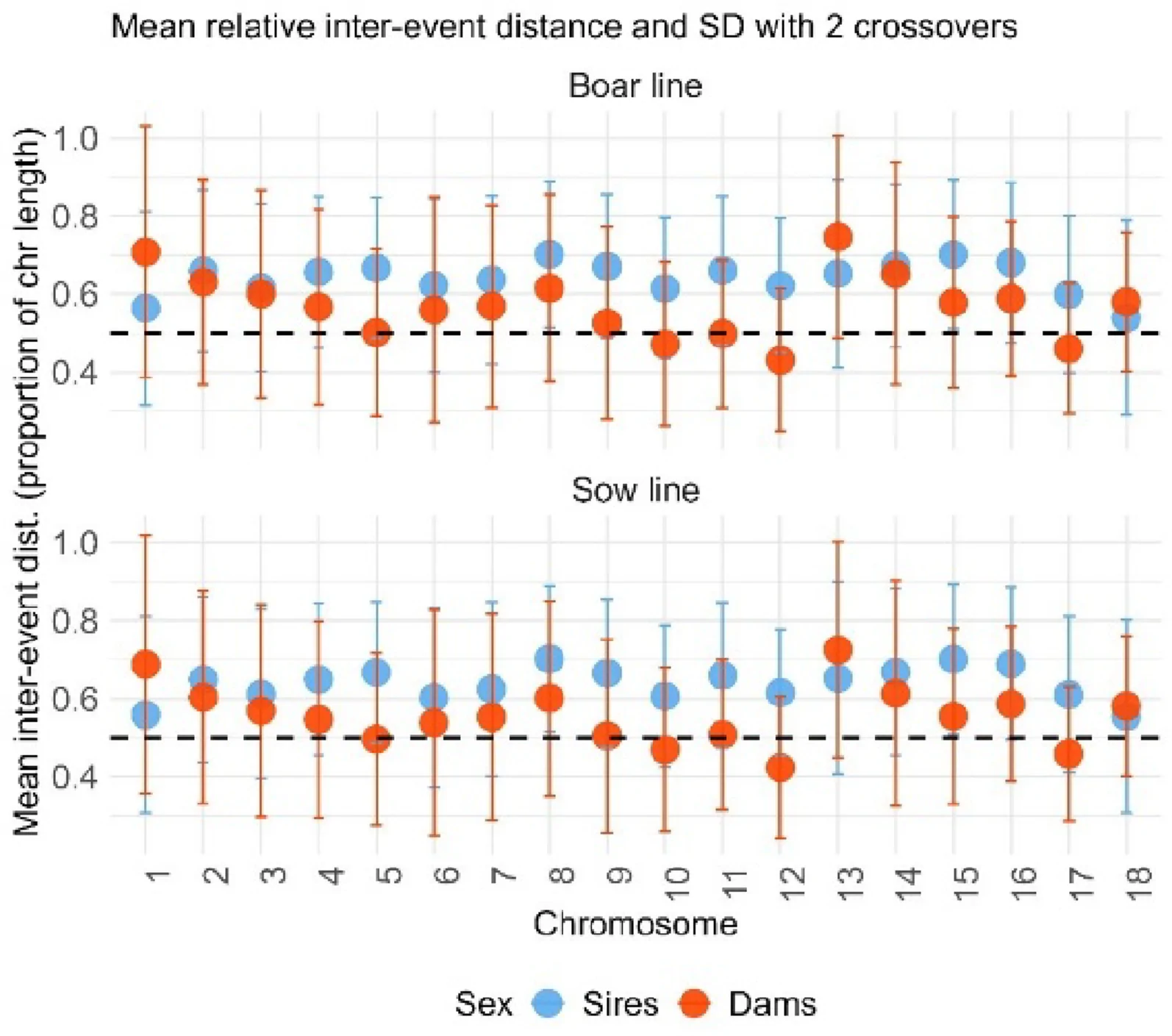
Mean inter-event distances as proportion of total chromosome length and SD with two CO per chromosome, line and sex. A linear model including sex, chromosome, and their interaction were fitted to assess the differences in inter-event distances between sexes. This was done for each line separately. For both lines, a highly significant sex-by-chromosome interaction was observed (*p* < 2e-16), with all individual chromosome × sex interactions being significant, indicating that sex differences in inter-event distance vary across chromosomes. The dashed black line indicates ½ each chromosome’s length

To investigate the relationship between inter-event distance and intrachromosomal genetic shuffling, we estimated the mean inter-event distance for chromosomes with two crossovers per chromosome, line and sex. This measure might allow us to distinguish between crossover configurations that may result in similar intrachromosomal genetic shuffling values but differ in their crossover positioning. Particularly for 2 crossovers per chromosome, intrachromosomal shuffling is highest when the distance between crossovers is ½ of the chromosome’s length. Figure 6 shows the mean inter-event distance between crossovers as a proportion of total chromosome length in parent-offspring pairs in chromosomes with two crossovers, distinguished by sex. In line with the results for intrachromosomal shuffling, sires showed, on average, greater mean inter-event distance between crossovers than dams, except for SSC1, 13, and 18. For SSC1 and 13, sires exhibited lower mean inter-event distance (Fig. 6) but higher intrachromosomal genetic shuffling (Fig. 5) than dams. While for SSC 18, sires exhibited slightly lower mean-inter event distance (Fig. 6) and lower intrachromosomal genetic shuffling (Fig. 5) than dams. These results might suggest that for SSC1 and 13, sires tend to localize crossovers more evenly spaced than dams, which leads to increased intrachromosomal genetic shuffling [24]. For SSC18, sires tend to localize crossovers closer between each other compared to dams, which leads to lower intrachromosomal genetic shuffling [24].

### Detailed analysis for SSC 5

For SSC 5 there was a peak in recombination rate between the two SNPs positioned at 11.8556 Mb and 12.1131 Mb in both lines. The peak in recombination rate could be a result of a mapping error in the reference genome, or an insertion or deletion compared to the reference genome. We investigated the Sscrofa11.1 reference genome to examine the region surrounding the recombination peak and observed a discrepancy with the SNP map file. Specifically, the region between the two flanking SNPs appears to be missing in the assembled chromosome. According to the NCBI database (https://www.ncbi.nlm.nih.gov/gene/100624208), this region is located within an unplaced scaffold, NW_018084968.1, suggesting a potential misassembly or unresolved genomic region in the reference. Thus, the peak of recombination between both SNPs reported by LINKPHASE3 might reflect the underestimation of the physical distance between them in the used map. We decided to retain these SNPs on SSC5 to estimate the number of crossovers, but we did not use them to infer the physical distance between them, due to the potential discrepancy in reported map positions.

## Discussion

In this study we aimed to (1) estimate and compare autosomal recombination rates (cM/Mb), ACC, and intrachromosomal genetic shuffling between two pig lines with different selection histories; (2) explore differences in recombination landscapes between individuals with high and low crossover counts; and (3) analyze distances between consecutive crossover events in both lines. The overall mean ACC was 17.4 in sires and 22.4 in dams of the boar line, and 18.1 in sires and 23.9 in dams of the sow line, indicating that sires and dams from the sow line transmitted on average about 1 additional crossover than those from the boar line. On average, crossovers were more frequent towards the extreme of the chromosomes. For individuals with a high ACC, crossovers were relatively higher in center regions of multiple chromosomes than for individuals with a low ACC. For parents transmitting two crossovers on one chromosome, we found sires to have generally higher levels of intrachromosomal genetic shuffling than dams and higher inter-event distance between crossovers, with this pattern observed across most of the chromosomes. Altogether, our results provide insights into crossover patterns and the potential of optimizing breeding strategies by selection on crossover count.

### Sex-differences in recombination rate, ACC, and intrachromosomal genetic shuffling

In general, the results for ACC from our study are only slightly lower to those reported by Brekke et al. [15]. Brekke et al. [15] study used similar lines, but used a different software package to detect crossovers and twice as many SNPs, which can explain the slightly higher ACC values. As expected, results show the presence of heterochiasmy in both lines [6, 7, 15], with higher recombination rate in sows for all chromosomes. However, this pattern was inverted in the two chromosomes with the largest physical length (SSC1 and SSC13), which was also reported in a previous study in pigs [7]. Although sex differences in recombination rate are well studied in pigs, this observation suggests that heterochiasmy may not be uniform across the genome and its direction may depend on the length of the chromosome. Although the actual cause of heterochiasmy remains unknown, previous studies showed its presence in other species [1], and [5] reported evidence for different genetic architectures of recombination rate between sexes in species like cattle, sheep, pigs, chickens, and Atlantic salmon, indicating that different genes might be involved, thereby suggesting some level of evolutionary independence.

Our results also showed that sows have slightly, but significantly, lower intrachromosomal genetic shuffling than boars in only one line, which agrees with a previous study in pigs [29]. One possible cause of this might be that females have a higher recombination rate towards the extreme chromosome regions than sires [7, 15], and crossovers localized in the extremes of the chromosomes are known to produce less shuffling than those localized in the center regions [5, 24]. Therefore, this difference in recombination landscape between sexes might be one possible explanation for the highest intrachromosomal shuffling being observed in males [29]. Another possible explanation might be the previously observed higher strength of crossing-over interference in boars compared to sows reported by Brekke et al., 2025 [24, 25], , which tends to increase genetic shuffling [5, 24].

For both lines and sexes and on every chromosome, parents transmitted most frequently only 1 crossover to their offspring (Fig. 2), and in > 93% of the cases, either 0, 1, or 2 crossovers were transmitted. However, dams transmitted more frequently 3 or more CO than sires in both lines, except for SSC 1 and 13, which are the longest chromosomes. This higher frequency of transmitting 3 or more crossovers in dams could be due to stronger crossing-over interference [24], as previously reported in sires compared to dams in pigs [24, 25].

### Differences in ACC between the pig lines

As mentioned, both very low and very high crossover count may impair fertility [5, 17–19], suggesting that there might be an optimal crossover count for fertility performance. Our results indicate that parents of sow line transmitted nearly one additional crossover in both sexes compared to the boar line (Table 2), and a higher proportion of sow parents transmitted more than 2 crossovers than of boar parents (Fig. 2). The suggests that the difference in selection histories between the pig lines of our study might have led to an indirect selection on ACC resulting in one additional crossover in the sow line compared to the boar line.

### Differences in the recombination landscape between the top and bottom 10% of parent-offspring pairs

On average, individuals exhibited a higher crossover frequency in the extreme chromosome regions compared to the center regions. This pattern of crossovers was already previously reported in pigs [6, 7], and can be explained by differences in chromatin structure along the chromosome. In the central regions, heterochromatin is more condensed, resulting in a lower probability of crossovers [1]. In contrast with other species [30, 31], the centromeric region does not seem to influence the crossover distribution across chromosomes in pigs, which might explain the overall similar recombination landscape between acrocentric and non-acrocentric chromosomes in the pig [6].

Interestingly, our results also showed that individuals with the highest ACC had relatively slightly more crossovers localized in the center regions of the chromosomes (middle 50% of the chromosome) than those with the lowest ACC. This effect was more pronounced in dams. These results suggest that if we can increase ACC with selection, we might also increase the occurrence of crossovers in the center regions of the chromosomes. Previous studies have identified several significant SNPs in the center regions of chromosomes linked to important breeding goal traits, such as fertility and productivity traits in pigs [32–34]. These findings might indicate that increasing crossover count in central regions that normally have low crossover count may have relevant implications for breeding. However, further analyses are required to quantify whether such increases would result in the release of additive genetic variance.

It is important to mention that our analyses reveal broad patterns of recombination landscapes along chromosomes, but do not resolve fine-scale features such as hotspots. Pigs can carry a functional PRDM9 gene [7], which may direct hotspot activity, however the low-density SNP chip used here limits our ability to detect such fine-scale variation.

### Intrachromosomal genetic shuffling and inter-event distance

Although recombination rate (i.e., the number of crossovers) is usually the parameter considered when studying the potential use of recombination for breeding, the effect of each crossover depends on where it occurs. Intrachromosomal genetic shuffling estimates the degree of information exchange between chromosomes, based on the position of the crossovers. Shuffling is maximized when each chromosome contains 50% of each paternal (or maternal) grandparent’s information. Hence, with one crossover, shuffling is maximized when the crossover occurs at the chromosome center and decreases toward the ends [24]. With two crossovers, shuffling is maximized when the distance between the crossovers is equal to ½ the chromosome’s length, suggesting that shuffling is expected to behave quadratically with the inter-event distance between the two crossovers. Our results show that individuals differed in the inter-event distance and level of genetic shuffling. On most chromosomes, the average inter-event distances exceeded ½ of the chromosome length, while the inter-event distance was lower and intrachromosomal shuffling higher for sires than for dams. Together, the patterns of intrachromosomal genetic shuffling and inter-event distance may underscore sex-specific differences in crossover positioning when two crossovers occur on the same chromosome.

We only analyzed the inter-event distances for chromosomes with two crossovers, as it was the most common transmission category for studying distances in our results. To our knowledge the heritability of the inter-event distance is still unexplored in pigs, but a study in cattle reported low but non-zero heritabilities [35]. Therefore, studying the inter-event distance could offer valuable insights for breeding. Most breeding goal traits are complex and influenced by many genes, although the exact locations of the genes remain largely unknown. If polygenes are uniformly distributed along the chromosome, selecting for increased intrachromosomal shuffling may promote a more uniform release of additive genetic variance across the chromosome. However, if polygenes are clustered or the aim is to release additive genetic variance within a specific region, it could be useful to also select for short inter-event distances, because short inter-event distances might break up linkage disequilibrium more effectively, thereby releasing more additive genetic variance. Thus, understanding inter-event distances may improve breeding strategies by promoting additive genetic variance release and more efficient purifying selection.

### Crossing-over interference

Beyond the analysis of inter-event distances, it would be interesting in future work to explore their relationship with crossover interference, which was previously reported to be positive in pigs and to differ between sexes [25]. While positive interference is generally associated with larger distances between consecutive crossovers, the exact relationship may not be straightforward, as the underlying biological mechanisms are still not fully understood [5].

Our results show that the top 10% of parent-offspring pairs with the highest ACC exhibited ~ 65% higher intrachromosomal genetic shuffling than the bottom 10%. When the number of crossovers increases, crossovers tend to be more evenly spaced across the genome, which enhances intrachromosomal shuffling. This may reflect a stronger crossing-over interference in the animals with the highest ACC. Although the mechanisms behind crossing-over interference remain not well understood, Veller et al. [24] proposed that crossing-over interference may be an adaptive function, as it increases genetic shuffling by preventing crossovers from occurring too close to each other. Therefore, interference might play a role in maximizing intrachromosomal genetic shuffling. This suggests that individuals with higher numbers of crossovers may also exhibit stronger positive crossing-over interference [41], thereby contributing to a higher shuffling.

### Potential selection response for ACC

Selecting for the ACC may be advantageous, as it could promote the release of additive genetic variance in breeding goal traits. Based on the breeders equation [36] and assuming a heritability of 0.07 across sexes [7, 29], we can estimate that the potential genetic gain based on mass selection when selecting the top 5% of individuals with the highest ACC in the sow line (in our study, the mean of that group was ~ 32 ACC) is 3.8% compared to the overall mean of 20.9 ACC. Therefore, there might be potential to change ACC in breeding programs. Previous studies [7, 15], however, concluded that selection for ACC in pigs may have limited practical utility, due to the low heritability, the physiological limits associated with very high ACC, and the large change in ACC that is expected to be needed to substantially increase genetic variation in other traits. Given that those conclusions relied on several expectations, more research is needed to empirically investigate the relationship between ACC and genetic variation, and determine whether ACC is the best parameter in this context. Our work, for example, suggests that also considering intrachromosomal genetic shuffling and inter-event distance between crossovers might release more genetic variance than focusing only on ACC. Moreover, it would be relevant to further study the relationship between ACC and genetic variation of important breeding goal traits.

#### Recombination rate versus map file

Recombination rate depends on the physical distance between SNPs. During the crossover identification, we found some regions where the recombination rate was not in accordance with the physical distance between SNPs, but generally, we could solve that by removing conflicting SNPs from our map files. However, for one region on SSC5, this was not the case, and a detailed analysis showed that the physical distance was likely underestimated in the map file. We are not aware of previous studies reporting the same issue in this region. However, those observations indicate that the recombination pattern can help to detect errors in the map file.

## Conclusions

We studied the crossover patterns in a sow and boar line, and observed that the dams and sires from the sow line transmitted on average one more crossover than the boar line, likely due to different selection histories. In both lines, dams transmitted more crossovers than sires. Although sires transmitted fewer crossovers, they showed higher overall intrachromosomal genetic shuffling, indicating a more even crossover distribution across the genome. We also found that individuals transmitting more crossovers had relatively slightly more crossovers towards the center regions of the chromosomes. Additionally, we analyzed distances between consecutive crossovers when two crossovers were transmitted per chromosome. Altogether, our results show that it could be beneficial to not only focus on crossover count to increase the additive genetic variance of breeding goal traits, but to also include selection on intrachromosomal shuffling and the inter-event distance, as together they more effectively capture the spatial distribution of crossovers.

## Supplementary Information

Below is the link to the electronic supplementary material.

Supplementary Material 1

Supplementary Material 2

## Data Availability

The data supporting the findings of this study are available from Topigs Norsvin. However, restrictions apply, as these data were used under license for the current study and are not publicly available.
