# Supplementary material for: Determining crossover count and position in two pig lines with different selection histories
Source: Genet Sel Evol. 2026 Jun 20;58:43. doi: 10.1186/s12711-026-01060-x (PMC13491639; doi:10.1186/s12711-026-01060-x)
Supplement: Supplementary file 1 [file 12711_2026_1060_MOESM1_ESM.docx]

**Additional file 1**

Figure S1: Flowchart of selection of target individuals in both lines.

Figure S2: Steps to estimate ACC on the parent-offspring pairs of both pig lines.

Figure S3: Observed crossover count per chromosome and expected by a Poisson distribution in both lines.

Figure S4: Distribution of ACC in both lines and sexes.

Figure S5: Recombination landscape of all autosomes for top and bottom 10% of sires (Sow line)

Figure S6: Recombination landscape of all autosomes for top and bottom 10% of dams (Sow line)

Figure S7: Recombination landscape of all autosomes for top and bottom 10% of sires (Boar line)

Figure S8: Recombination landscape of all autosomes for top and bottom 10% of dams (Boar line)

Figure S9: Recombination landscape for the subgroup of individuals within the cloud of dots of Fig. 6 and for all individuals (Sow line)

Figure S10: Recombination landscape for the subgroup of individuals within the cloud of dots of Fig. 6 and for all individuals (Boar line)

Fig. S11: Mean Intrachromosomal Genetic Shuffling by chromosome, sex and line when the number of transmitted crossovers per chromosome (CC) is 3
