# Supplementary material for: Determining crossover count and position in two pig lines with different selection histories
Source: Genet Sel Evol. 2026 Jun 20;58:43. doi: 10.1186/s12711-026-01060-x (PMC13491639; doi:10.1186/s12711-026-01060-x)
Supplement: Supplementary file 2 [file 12711_2026_1060_MOESM2_ESM.docx]

**Additional File 2**

**Table S1. Recombination rates (cM/Mb) for autosomal chromosomes in both lines and sexes.**

|  | **Number of SNPs** | |  | **Sires (Boar line)** | | **Dams (Boar line)** | | **Sires (Sow line)** | | **Dams (Sow line)** | |
| --- | --- | --- | --- | --- | --- | --- | --- | --- | --- | --- | --- |
| **SSC** | **Boar Line** | **Sow Line** | **Physical Length (Mb)** | **Genetic Length (cM)** | **Recombination rate**  **(cM/Mb)** | **Genetic Length (cM)** | **cM/Mb** | **Genetic Length (cM)** | **Recombination rate**  **(cM/Mb)** | **Genetic Length (cM)** | **cM/Mb** |
| 1 | 1887 | 1887 | 274.3 | 159.8 | 0.58 | 136.8 | 0.50 | 162.4 | 0.59 | 142.1 | 0.52 |
| 2 | 1381 | 1363 | 151.9 | 113.5 | 0.75 | 136.5 | 0.90 | 116.3 | 0.77 | 147.2 | 0.97 |
| 3 | 1154 | 1147 | 132.8 | 120.1 | 0.90 | 144.2 | 1.09 | 122.5 | 0.92 | 158.2 | 1.19 |
| 4 | 1385 | 1372 | 130.9 | 109.6 | 0.84 | 145.4 | 1.11 | 111.0 | 0.85 | 153.8 | 1.17 |
| 5 | 1017 | 1007 | 104.5 | 105.8 | 1.01 | 152.6 | 1.46 | 106.7 | 1.02 | 156.7 | 1.50 |
| 6 | 1606 | 1599 | 170.8 | 128.5 | 0.75 | 170.4 | 1.00 | 133.8 | 0.78 | 183.5 | 1.07 |
| 7 | 1188 | 1177 | 121.8 | 117.1 | 0.96 | 152.5 | 1.25 | 120.4 | 0.99 | 159.9 | 1.31 |
| 8 | 1135 | 1131 | 139.0 | 107.6 | 0.77 | 134.6 | 0.97 | 107.3 | 0.77 | 139.7 | 1.01 |
| 9 | 1369 | 1366 | 139.5 | 109.1 | 0.78 | 162.1 | 1.16 | 110.3 | 0.79 | 169.2 | 1.21 |
| 10 | 761 | 756 | 69.4 | 96.5 | 1.39 | 135.3 | 1.95 | 100.0 | 1.44 | 140.1 | 2.02 |
| 11 | 775 | 763 | 79.2 | 77.2 | 0.98 | 120.1 | 1.52 | 79.7 | 1.01 | 123.8 | 1.56 |
| 12 | 533 | 530 | 61.6 | 83.4 | 1.35 | 132.3 | 2.15 | 88.5 | 1.44 | 139.3 | 2.26 |
| 13 | 1598 | 1571 | 208.3 | 130.1 | 0.62 | 121.2 | 0.58 | 130.9 | 0.63 | 124.5 | 0.60 |
| 14 | 1496 | 1493 | 141.8 | 114.2 | 0.81 | 142.9 | 1.01 | 115.6 | 0.82 | 152.7 | 1.08 |
| 15 | 1238 | 1236 | 140.4 | 107.4 | 0.76 | 123.4 | 0.88 | 106.4 | 0.76 | 125.7 | 0.90 |
| 16 | 930 | 929 | 79.9 | 74.3 | 0.93 | 112.1 | 1.40 | 77.8 | 0.97 | 112.7 | 1.41 |
| 17 | 547 | 545 | 63.5 | 66.2 | 1.04 | 114.8 | 1.81 | 68.1 | 1.07 | 119.5 | 1.88 |
| 18 | 541 | 543 | 56.0 | 58.3 | 1.04 | 96.2 | 1.72 | 59.1 | 1.06 | 101.1 | 1.81 |
| Total | 20,541 | 20,415 | 2,265.6 | 1,878.6 | 0.90 | 2,433.2 | 1.25 | 1,916.7 | 0.93 | 2,549.6 | 1.30 |

**Table S2. Observed mean crossover count for each chromosome by line and sex.**

|  |  | **Observed mean crossover count** | | | |
| --- | --- | --- | --- | --- | --- |
| **Chromosome** | **Physical Length (Mb)** | **Boar line** | | **Sow line** | |
|  |  | **Sires** | **Dams** | **Sires** | **Dams** |
| 1 | 274.3 | 1.5 | 1.3 | 1.6 | 1.3 |
| 2 | 151.9 | 1.0 | 1.2 | 1.1 | 1.3 |
| 3 | 132.8 | 1.1 | 1.3 | 1.2 | 1.5 |
| 4 | 130.9 | 1.0 | 1.3 | 1.1 | 1.4 |
| 5 | 104.5 | 1.0 | 1.4 | 1.0 | 1.5 |
| 6 | 170.8 | 1.2 | 1.6 | 1.3 | 1.7 |
| 7 | 121.8 | 1.1 | 1.4 | 1.2 | 1.5 |
| 8 | 139.0 | 1.0 | 1.3 | 1.0 | 1.3 |
| 9 | 139.5 | 1.0 | 1.5 | 1.0 | 1.6 |
| 10 | 69.4 | 0.9 | 1.3 | 0.9 | 1.3 |
| 11 | 79.2 | 0.7 | 1.1 | 0.8 | 1.2 |
| 12 | 61.6 | 0.8 | 1.2 | 0.8 | 1.2 |
| 13 | 208.3 | 1.2 | 1.1 | 1.2 | 1.2 |
| 14 | 141.8 | 1.1 | 1.3 | 1.1 | 1.4 |
| 15 | 140.4 | 1.0 | 1.1 | 1.0 | 1.2 |
| 16 | 79.9 | 0.7 | 1.0 | 0.7 | 1.1 |
| 17 | 63.5 | 0.6 | 1.0 | 0.6 | 1.1 |
| 18 | 56.0 | 0.5 | 0.9 | 0.5 | 0.9 |

^a^ The relationship between crossover count and chromosome length was analyzed using a linear model including chromosome length (Mb), sex, line, and their interactions. Interactions of chromosome length with sex and line were highly significant (P < 2 × 10⁻¹⁶), and the three-way interaction was also significant (P = 0.026), indicating differences in slope among sex–line groups. Slopes for each sex × line combination (crossovers/Mb, 95% CI) were: boar line sires 0.00394 (0.00392–0.00397), boar line dams 0.00120 (0.00119–0.00121), sow line sires 0.00411 (0.00409–0.00414), and sow line dams 0.00144 (0.00143–0.00145).

**Table S3. Proportion of distal and central crossovers in both lines and sexes**

| Chr | Sires (Sow line) | | | | Dams (Sow line) | | | | Sires (Boar line) | | | | Dams (Boar line) | | | |
| --- | --- | --- | --- | --- | --- | --- | --- | --- | --- | --- | --- | --- | --- | --- | --- | --- |
|  | Top | | Bottom | | Top | | Bottom | | Top | | Bottom | | Top | | Bottom | |
|  | Distal | Central | Distal | Central | Distal | Central | Distal | Central | Distal | Central | Distal | Central | Distal | Central | Distal | Central |
| 1 | 0.68 | 0.32* | 0.72 | 0.28* | 0.93 | 0.07* | 0.97 | 0.03* | 0.69 | 0.31* | 0.73 | 0.27* | 0.94 | 0.06* | 0.98 | 0.02* |
| 2 | 0.81 | 0.19* | 0.85 | 0.15* | 0.82 | 0.18* | 0.91 | 0.09* | 0.82 | 0.18* | 0.86 | 0.14* | 0.84 | 0.16* | 0.95 | 0.05* |
| 3 | 0.73 | 0.27* | 0.76 | 0.24* | 0.78 | 0.22* | 0.89 | 0.11* | 0.73 | 0.27* | 0.77 | 0.23* | 0.79 | 0.21* | 0.91 | 0.09* |
| 4 | 0.78 | 0.22* | 0.80 | 0.20* | 0.67 | 0.33* | 0.79 | 0.21* | 0.80 | 0.20* | 0.82 | 0.18* | 0.69 | 0.31* | 0.85 | 0.15* |
| 5 | 0.80 | 0.20 | 0.81 | 0.19 | 0.64 | 0.36* | 0.68 | 0.32* | 0.80 | 0.20 | 0.81 | 0.19 | 0.63 | 0.37* | 0.68 | 0.32* |
| 6 | 0.73 | 0.27* | 0.78 | 0.22* | 0.77 | 0.23* | 0.86 | 0.14* | 0.76 | 0.24* | 0.81 | 0.19* | 0.81 | 0.19* | 0.90 | 0.10* |
| 7 | 0.75 | 0.25* | 0.80 | 0.20* | 0.73 | 0.27* | 0.80 | 0.20* | 0.76 | 0.24* | 0.81 | 0.19* | 0.71 | 0.29* | 0.82 | 0.18* |
| 8 | 0.86 | 0.14* | 0.88 | 0.12* | 0.75 | 0.25* | 0.87 | 0.13* | 0.87 | 0.13 | 0.88 | 0.12 | 0.74 | 0.26* | 0.89 | 0.11* |
| 9 | 0.82 | 0.18* | 0.85 | 0.15* | 0.61 | 0.39* | 0.69 | 0.31* | 0.84 | 0.16 | 0.84 | 0.16 | 0.61 | 0.39* | 0.72 | 0.28* |
| 10 | 0.71 | 0.29 | 0.67 | 0.33 | 0.61 | 0.39* | 0.63 | 0.37* | 0.71 | 0.29 | 0.69 | 0.31 | 0.49 | 0.51* | 0.62 | 0.38* |
| 11 | 0.77 | 0.23 | 0.76 | 0.24 | 0.52 | 0.48* | 0.58 | 0.42* | 0.77 | 0.23 | 0.75 | 0.25 | 0.49 | 0.51* | 0.56 | 0.44* |
| 12 | 0.71 | 0.29 | 0.67 | 0.33 | 0.44 | 0.56* | 0.47 | 0.53* | 0.72 | 0.28 | 0.68 | 0.32 | 0.42 | 0.58* | 0.50 | 0.50* |
| 13 | 0.76 | 0.24* | 0.81 | 0.19* | 0.87 | 0.13* | 0.95 | 0.05* | 0.77 | 0.23* | 0.81 | 0.19* | 0.90 | 0.10* | 0.96 | 0.04* |
| 14 | 0.80 | 0.20* | 0.84 | 0.16* | 0.80 | 0.20* | 0.89 | 0.11* | 0.80 | 0.20* | 0.83 | 0.17* | 0.83 | 0.17* | 0.93 | 0.07* |
| 15 | 0.83 | 0.17* | 0.84 | 0.16* | 0.73 | 0.27* | 0.82 | 0.18* | 0.84 | 0.16 | 0.85 | 0.15 | 0.75 | 0.25* | 0.86 | 0.14* |
| 16 | 0.79 | 0.21 | 0.76 | 0.24 | 0.60 | 0.40* | 0.64 | 0.36* | 0.80 | 0.20 | 0.77 | 0.23 | 0.61 | 0.39* | 0.64 | 0.36* |
| 17 | 0.71 | 0.29 | 0.69 | 0.31 | 0.55 | 0.45 | 0.55 | 0.45 | 0.71 | 0.29 | 0.70 | 0.30 | 0.54 | 0.46 | 0.52 | 0.48 |
| 18 | 0.69 | 0.31 | 0.65 | 0.35 | 0.67 | 0.33* | 0.69 | 0.31* | 0.66 | 0.34 | 0.63 | 0.37 | 0.68 | 0.32* | 0.71 | 0.29* |

^b^ One-sided Z-tests for differences in proportions (α = 0.05) were performed separately for each chromosome to test whether the top 10% had higher central crossover proportions and the bottom 10% higher distal crossover proportions

^*^ Indicate statistically significant proportions between top and bottom 10% groups

**Table S4. Estimated marginal means for intrachromosomal genetic shuffling for top and bottom 10% of individuals, by line and sex.**

| **Line** | **Sex** | **Group** | **EMM intrachr. Shuffling (SE)** | **95% CI** |
| --- | --- | --- | --- | --- |
| Sow | Sires | Bottom10 | 0.0136 (<0.0001) | 0.0135–0.0137 |
| Sow | Sires | Top10 | 0.0220 (<0.0001) | 0.0219–0.0221 |
| Sow | Dams | Bottom10 | 0.0134 (<0.0001) | 0.0133–0.0134 |
| Sow | Dams | Top10 | 0.0222 (<0.0001) | 0.0221–0.0223 |
| Boar | Sires | Bottom10 | 0.0134 (<0.0001) | 0.0133–0.0134 |
| Boar | Sires | Top10 | 0.0217 (<0.0001) | 0.0216–0.0218 |
| Boar | Dams | Bottom10 | 0.0127 (<0.0001) | 0.0126–0.0128 |
| Boar | Dams | Top10 | 0.0219 (<0.0001) | 0.0218–0.0220 |

^c^) Linear models were used to compare intrachromosomal shuffling between top and bottom 10% groups. Group (top/bottom 10%), line, sex, and their interactions were included in the model. Top 10% individuals showed higher shuffling than bottom 10% (p < 0.001), and a significant group × line × sex interaction was detected (p = 0.0025).
